# *Disclisioproctaedmondsii* (Butler, 1882) comb. nov. (Lepidoptera, Geometridae, Larentiinae)

**DOI:** 10.3897/BDJ.11.e98935

**Published:** 2023-01-11

**Authors:** Héctor A. Vargas

**Affiliations:** 1 Universidad de Tarapacá, Facultad de Ciencias Agronómicas, Departamento de Recursos Ambientales, Arica, Chile Universidad de Tarapacá, Facultad de Ciencias Agronómicas, Departamento de Recursos Ambientales Arica Chile

**Keywords:** DNA barcoding, genitalia morphology, geometrid moths, Neotropical

## Abstract

**Background:**

The generic assignment of the geometrid moth *Xanthorhoeedmondsii* (Butler, 1882) (Lepidoptera, Geometridae, Larentiinae), originally described under *Hypochroma* Guenée, [1858], a junior homonym of *Hypochroma* Herrich-Schäffer, [1855] (Geometridae, Ennominae), is assessed using genitalia morphology and analysis of mitochondrial DNA sequences.

**New information:**

Morphological characters revealed closeness to the type species of *Disclisioprocta* Wallengren, 1861 (Larentiinae). In agreement with morphology, the molecular analysis clustered *X.edmondsii* with species of *Disclisioprocta* in a well-supported monophyletic group distantly related to members of *Xanthorhoe* Hübner, [1825]. Accordingly, *Disclisioproctaedmondsii* (Butler, 1882) comb. nov. is proposed.

## Introduction

Natural environments of South America harbour a high diversity of geometrid moths (Lepidoptera, Geometridae), whose taxonomy remains insufficiently studied (Hausmann and Parra 2009, Brehm et al. 2016, Murillo-Ramos et al. 2021). Besides the frequent discovery of new species (Brehm 2018, Ramos-González et al. 2019, Moraes et al. 2021, Vargas 2021), generic assignments of most of the already described species deserve assessment, as suggested by new combinations arising in taxonomic revisions (Parra 2018) and molecular phylogenetic analyses (Brehm et al. 2019).

The geometrid moth *Xanthorhoeedmondsii* (Butler, 1882) (Geometridae, Larentiinae) is known from Chile and Argentina (Butler 1882, Chalup 2014). It was originally described under *Hypochroma* Guenée, [1858], a junior homonym of *Hypochroma* Herrich-Schäffer, [1855] (Geometridae, Ennominae) (Parsons et al. 1999). Individuals from northern Chile reared from larvae collected on the ornamental plant *Bougainvilleaglabra* Choisy (Nyctaginaceae) were misidentified as *Chrismopteryxundularia* (Blanchard, 1852), based on comparison with material from central Chile deposited in the Museo Nacional de Historia Natural de Santiago (Vargas et al. 2010). Subsequent comparison with a photo of the type material deposited in the Natural History Museum, London, UK, allowed concluding that the specimens examined by Vargas et al. (2010) belong to *X.edmondsii*.

Morphological characters of the genitalia of geometrid moths are extremely useful in generic assignments (Pitkin 2002, Parra 2018, Viidalepp and Lindt 2019), which can be reinforced by phylogenetic analysis of DNA sequences (Brehm et al. 2019, Matson 2022, Wanke et al. 2022). An examination of the genitalia of *X.edmondsii* revealed remarkable morphological differences with *Xanthorhoemontanata* ([Denis & Schiffermüller], 1775), the type species of *Xanthorhoe* Hübner, [1825], suggesting instead closeness with *Disclisioproctastellata* (Guenée, [1858]), the type species of *Disclisioprocta* Wallengren, 1861. The aim of this study is to propose a new generic assignment for *X.edmondsii*, based on genitalia morphology and analysis of mitochondrial DNA sequences.

## Materials and methods

Specimens examined in this study were collected at light or reared from larvae collected on *B.glabra* in the Azapa Valley (18°31’16’’S, 70°10’42’’W), Arica Province, northern Chile. Photos of the genitalia were taken with a Leica Flexacam C1 digital camera attached to a Leica M125 stereomicroscope. Each image was constructed with about 5–10 photos assembled with the software Helicon Focus 8. The specimens of *X.edmondsii* are deposited in the “Colección Entomológica de la Universidad de Tarapacá” (IDEA), Arica, Chile. The specimens of *D.stellata* are deposited in the “Coleção Pe. Jesus de Santiago Moure (DZUP)”, Universidade Federal do Paraná, Curitiba, Paraná, Brazil.

Genomic DNA was extracted from two legs of a male adult using the QIAamp Fast DNA Tissue Kit, following the manufacturer’s instructions. DNA purification, PCR amplification and sequencing of the barcode region (Hebert et al. 2003) with the primers LCO1490 and HCO2198 (Folmer et al. 1994) were performed at Macrogen Inc. (Seoul, South Korea). The PCR programme was 5 min at 94°C, 35 cycles of 30 s at 94°C, 30 s at 47°C, 1 min at 72°C and a final elongation step of 10 min at 72°C. Analysis of this mitochondrial marker represents a helpful tool in generic assignments of Lepidoptera, including Geometridae (Wanke et al. 2019, Wanke et al. 2022, Vargas et al. 2022). The sequence of *X.edmondsii* was submitted to a Maximum Likelihood (ML) analysis with additional representatives of Larentiinae downloaded from BOLD (Ratnasingham and Hebert 2007), following the classification provided by Brehm et al. (2019) in which *Disclisioprocta* belongs to an unnamed clade sister to Euphyiini. The alignment included sequences of *Euphyia* Hübner, [1825] and *Oligopleura* Herrich-Schäffer, [1855] as representatives of this tribe, sequences of *Xanthorhoe* due to the current generic adscription of *X.edmondsii* (Parsons et al. 1999) and sequences of *Scotopteryx* Hübner, [1825] as outgroups. The software MEGA11 (Tamura et al. 2021) was used to perform sequence alignment with the ClustalW method and to determine genetic distance using the Kimura 2-Parameter (K2P) method. Before the ML analysis, the substitution saturation of the alignment was assessed with the Xia test, using the software DAMBE7 (Xia 2018). The ML analysis was performed using the software IQTREE 1.6.12 (Nguyen et al. 2014) in the web interface W-IQ-TREE (Trifinopoulos et al. 2016) with data partitioned to codon position. ModelFinder (Kalyaanamoorthy et al. 2017) selected TNe+I, F81+F and TN+F+G4 as the best fit models for 1st, 2nd and 3rd partitions, respectively. Branch support was assessed with 1000 replications of the Shimodaira-Hasegawa-like approximate likelihood ratio test (SH-aLRT, Guindon et al. (2010)) and ultrafast bootstrap (UFBoot, Hoang et al. (2017)). The unrooted tree was visualised in FigTree (Rambaut 2014) to root on *Scotopteryx*.

## Taxon treatments

### Disclisioprocta edmondsii

(Butler, 1882) comb. nov.

7F01F488-1D65-5433-9EE2-75152F1D8F2C

*Hypochromaedmondsii* Butler, 1882, p. 364. Angulo and Casanueva (1981), p. 21.
*Xanthorhoeedmondsii* (Butler, 1882): Parsons et al. (1999), p. 964.
*Chrismopteryxundularia* (Blanchard, 1852): Vargas et al. (2010), misidentification.

#### Materials

- **Type status:** Other material. **Occurrence:** individualCount: 5; occurrenceID: 99F2E982-FCB8-5FF0-B368-3F12C8EFF0A6; **Taxon:** scientificName: *Disclisioproctaedmondsii* (Butler, 1882); higherClassification: Insecta;Lepidoptera;Geometridae;Larentiinae; **Location:** continent: South America; country: Chile; stateProvince: Arica; locality: Azapa Valley; decimalLatitude: -18.52; decimalLongitude: -70.18; **Identification:** identifiedBy: Héctor A. Vargas; identificationRemarks: Genitalia slides HAV-1281, 1284, 1286, 1583, 1584; **Event:** samplingProtocol: Two males, three females emerged February 2006, reared fom larvae collected on Bougainvilleaglabra January 2006; **Record Level:** type: PhysicalObject; language: en; institutionCode: "Colección Entomológica de la Universidad de Tarapacá" (IDEA); basisOfRecord: "PreservedSpecimen"
- **Type status:** Other material. **Occurrence:** individualCount: 3; occurrenceID: 591A91A6-F832-5EFC-85C4-F03775D0D824; **Taxon:** scientificName: *Disclisioproctaedmondsii* (Butler, 1882); higherClassification: Insecta;Lepidoptera;Geometridae;Larentiinae; **Location:** continent: South America; country: Chile; stateProvince: Arica; locality: Azapa Valley; decimalLatitude: -18.52; decimalLongitude: -70.18; **Identification:** identifiedBy: Héctor A. Vargas; identificationRemarks: Genitalia slides HAV-1283, 1285, 1287; **Event:** samplingProtocol: Two males, one female September 2006 at light; **Record Level:** type: PhysicalObject; language: en; institutionCode: "Colección Entomológica de la Universidad de Tarapacá" (IDEA); basisOfRecord: "PreservedSpecimen"
- **Type status:** Other material. **Occurrence:** individualCount: 1; associatedSequences: BOLD Process ID GEONC001-22; occurrenceID: 6C800146-205F-5AA7-85A5-4470D74FCA88; **Taxon:** scientificName: *Disclisioproctaedmondsii* (Butler, 1882); higherClassification: Insecta;Lepidoptera;Geometridae;Larentiinae; **Location:** continent: South America; country: Chile; stateProvince: Arica; locality: Azapa Valley; decimalLatitude: -18.52; decimalLongitude: -70.18; **Identification:** identifiedBy: Héctor A. Vargas; identificationRemarks: Genitalia slide HAV-1580; **Event:** samplingProtocol: One male May 2022 at light; **Record Level:** type: PhysicalObject; language: en; institutionCode: "Colección Entomológica de la Universidad de Tarapacá" (IDEA); basisOfRecord: "PreservedSpecimen"

#### Description

**Figure 1.**
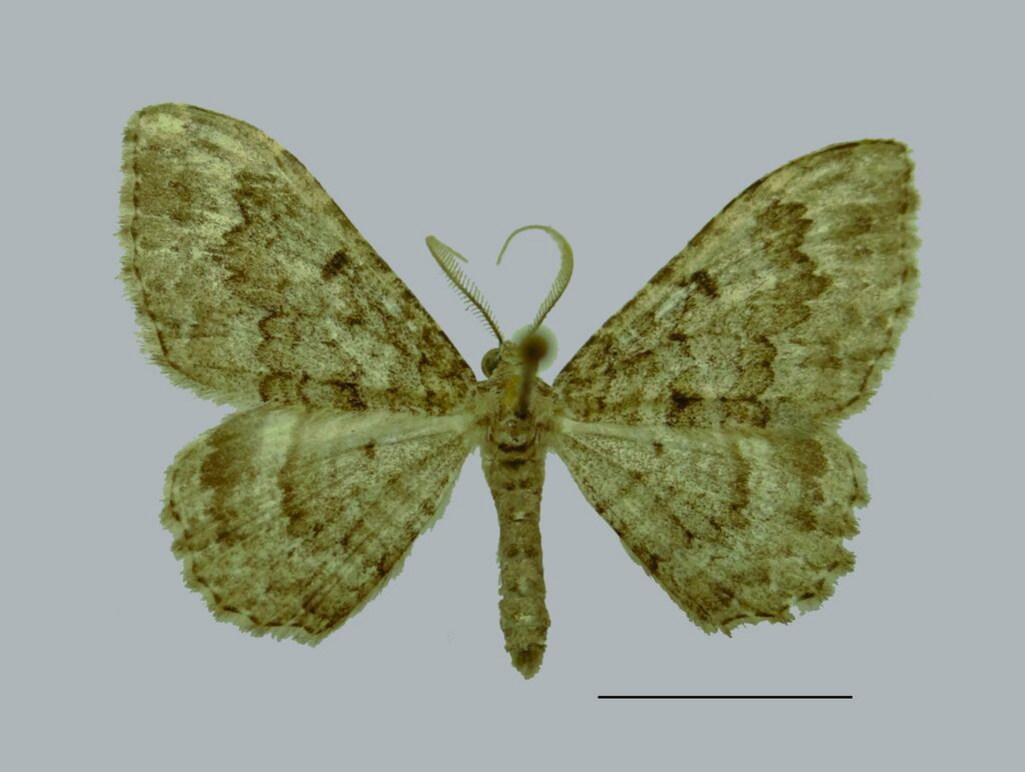
Male adult of *Disclisioproctaedmondsii* (Butler, 1882) comb. nov., dorsal view. Scale bar 10 mm.

Male habitus in Fig. 1. Although the male abdominal segments VII and VIII are not part of the genitalia, these are described here and illustrated because the morphology of the sclerites of these segments can be modified in different groups of Larentiinae (Viidalepp 2011).

**Figure 2.**
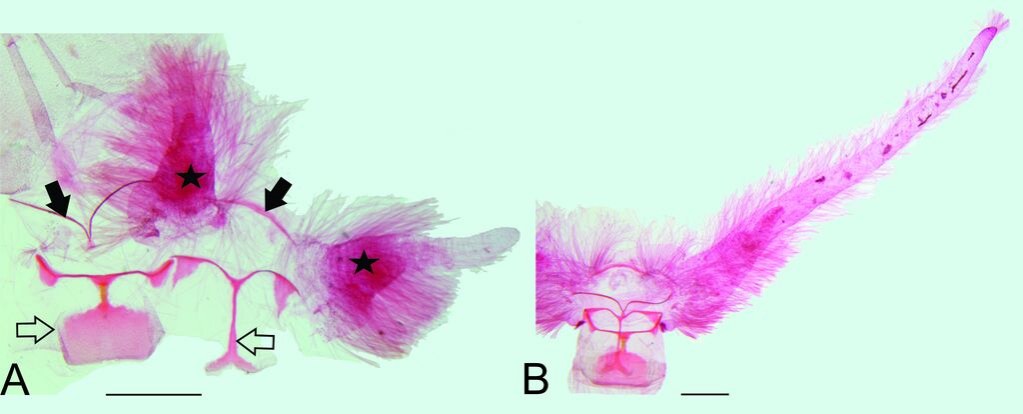
Male abdominal segments VII and VIII of *Disclisioproctaedmondsii* (Butler, 1882) comb. nov. A) Coremata (stars) of segment VII and sclerites of segments VII (closed arrows) and VIII (open arrows); terga on left, sterna on right. B) Segments VII and VIII showing right corema of segment VII expanded. Scale bar 1 mm.

Male abdominal segments VII and VIII (Fig. 2). Segment VII mostly membranous; tergum a transverse stripe strongly posteriorly folded in the middle; sternum a transverse stripe; pleura with pair of coremata. Segment VIII mostly membranous; tergum an anterior transverse stripe with semicircular expansion on tips, connected by a short longitudinal stripe with a posterior rectangular transverse plate; sternum an anterior transverse stripe posteriorly curved in the middle, projected as a narrow longitudinal stripe posteriorly bifid, triangular expansion near tip of the anterior transverse stripe.

**Figure 3.**
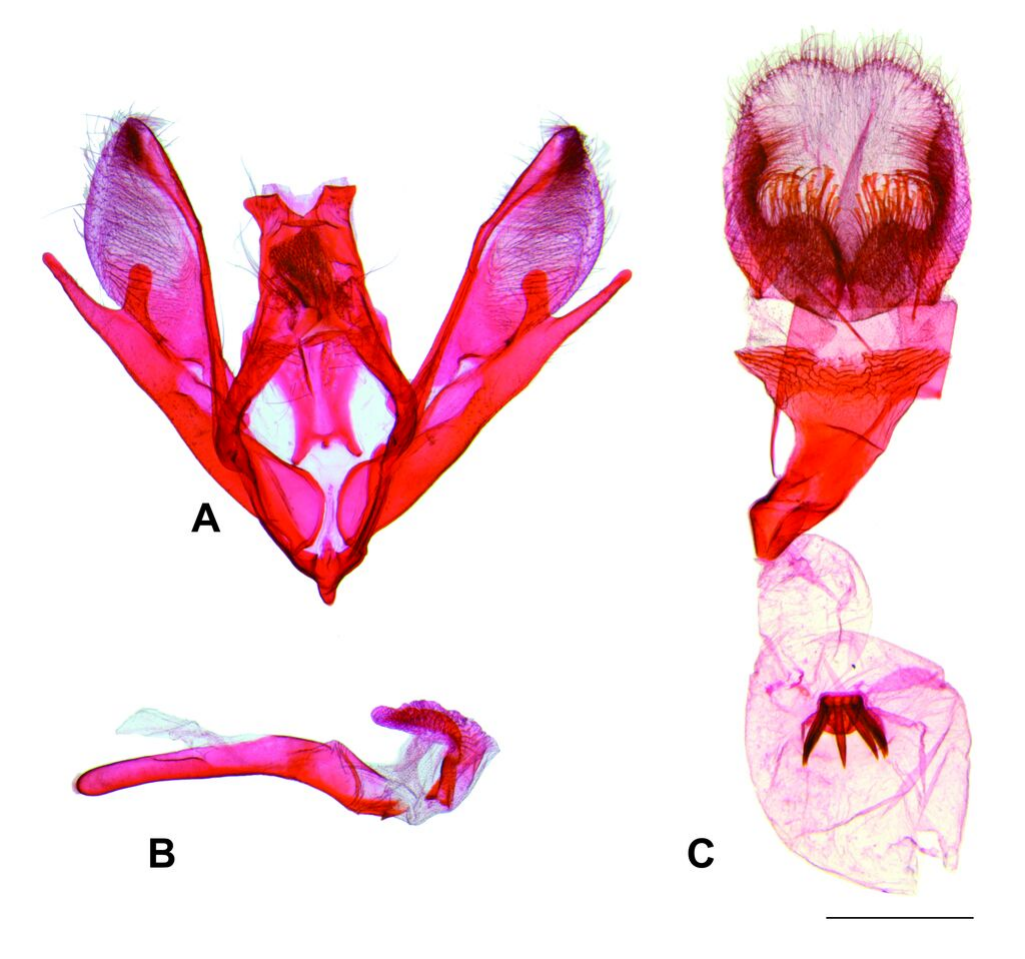
Genitalia of *Disclisioproctaedmondsii* (Butler, 1882) comb. nov. A) Male genitalia, phallus removed. B) Phallus. C) Female genitalia. Scale bar 1 mm.

Male genitalia (Fig. 3). Uncus bifid with broad posterior concavity in the middle, truncate points slightly down-curved. Saccus with small rounded anterior projection. Subscaphium slightly sclerotised. Labides with lobe-like tip bearing setae. Manica heavily spinose. Juxta trapezoidal, ventral half of lateral margin broadly concave, ventral margin broadly concave. Valva elongated; costal sclerotised band not reaching apex; cucullus mostly membranous on distal half with abundant setae; sacculus broad, well-sclerotised; sacculus projection stout, apex almost reaches that of the distal margin of the cucullus, with a broader, dorsally projected basal process. Phallus cylindrical, anterior half straight, posterior half curved, with a small spine-like projection ventrally on posterior tip; vesica mostly membranous with a plate-like cornutus.

Female genitalia (Fig. 3). Papillae anales membranous, lobe-like, fused dorsally, posterior edge with setae on dorsal and lateral parts and elongated, flattened scales on ventral part. Apophyses posteriores rod-shaped, narrow, slightly longer than apophyses anteriores. Antrum well-sclerotised, flattened, ventrally curved in the middle, progressively straightening anteriorly. Ductus bursae membranous, about 2/3 length of the antrum. Corpus bursae membranous, spherical, with 5–7 stout spine-like signa arising ventrally from the anterior margin of a semicircular slightly sclerotised plate. Ductus seminalis arising near the posterior tip of ductus bursae.

#### Molecular analysis

**Figure 4.**
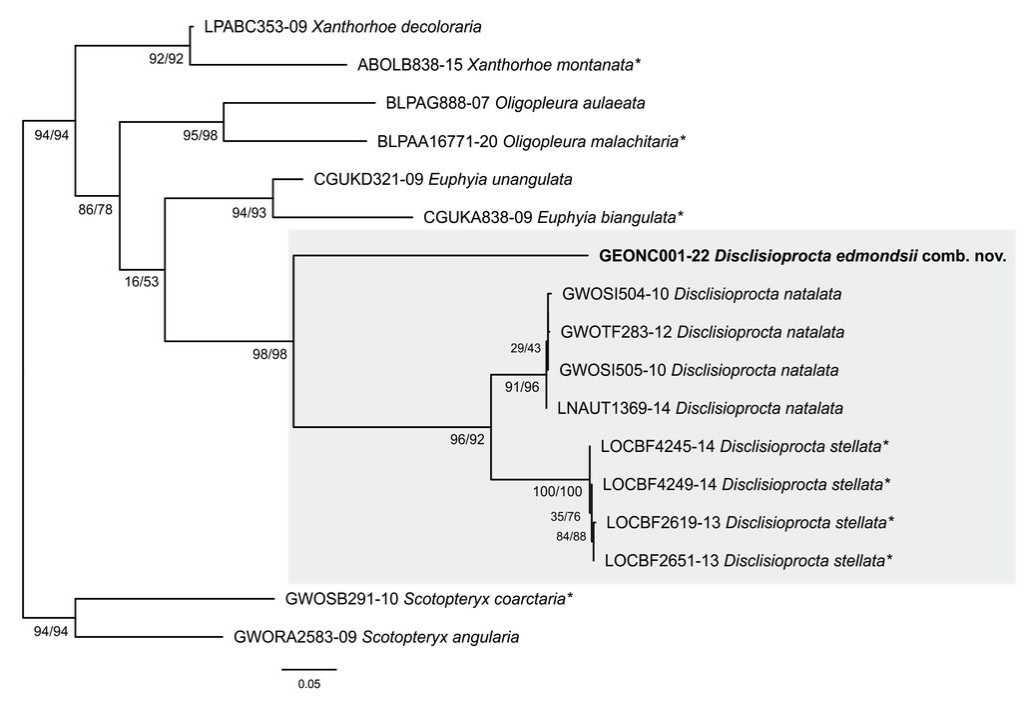
Maximum Likelihood tree of *Disclisioproctaedmondsii* (Butler, 1882) comb. nov. (bold) and representatives of the Euphyiini + Xanthorhoini complex of Larentiinae, based on mitochondrial DNA sequences. Grey rectangle indicates *Disclisioprocta* Wallengren, 1861. Asterisks indicate type species. Numbers indicate SH-aLRT/UFBoot values (1000 replicates).

Genetic distance of *D.edmondsii* (BOLD accession GEONC001-22) was 10.3–10.5% (K2P) with *D.natalata* and 11.0–11.5% with *D.stellata*, while the distance between the latter two was 6.2–7.1%. The alignment was suitable for phylogenetic analysis, as no evidence of stop codons was detected and the index of substitution saturation was smaller than the critical value (ISS < ISS.C; p < 0.001) in the Xia test. The ML analysis (Fig. 4) clustered (*D.edmondsii* (*D.natalata* + *D.stellata*)) with high support. Although each genus had reasonable statistical support in the ML analysis, relationships between genera were not resolved.

## Discussion

**Figure 5.**
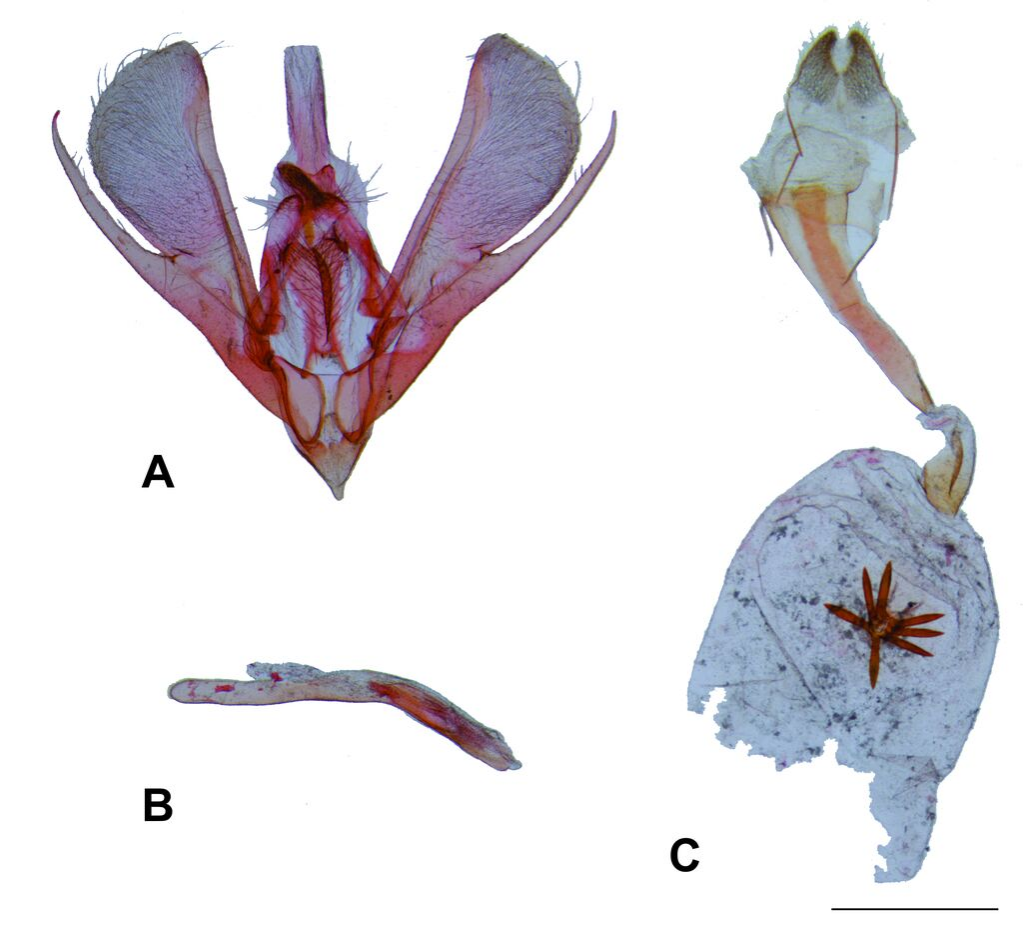
Genitalia of *Disclisioproctastellata* (Guenée, [1858]) from Brazil. **A** Male genitalia, phallus removed; **B** Phallus; **C** Female genitalia. Scale bar 1 mm.

Although the identification of synapomorphies for *Disclisioprocta* deserves further assessments based on better knowledge of the morphology of related genera, the morphological similarities between the genitalia of *D.edmondsii* (Fig. 3) and *D.stellata* (Fig. 5) provide support to consider them as congenerics. The two species have uncus bifid, costal sclerotised band not reaching apex of the cucullus, a stout sacculus projection and a plate-like cornutus in the male and a flattened elongated antrum and a cluster of elongated spine-like signa arising from a plate on the corpus bursae in the female. The same morphological characters support the removal of *D.edmondsii* from *Xanthorhoe*. The type species of this genus has an elongated rod-like uncus, costa as a sclerotised free arm extending beyond the cucullus apex, sacculus lacking a projection and numerous spine-like cornuti in the male and a short antrum and two elongated plate-like signa with small spines in the female (Expósito-Hermosa and Viidalepp 2011).

The result of the ML analysis is congruent with the genitalia morphology, since *D.edmondsii* clustered with two other representatives of *Disclisioprocta* in a well-supported clade, distantly related to members of *Xanthorhoe* (Fig. 4). The grouping of *D.stellata* and *D.natalata* as sister species agrees with the already highlighted remarkable morphological similarity of their genitalia (Hausmann 2009). The genetic distances of *D.edmondsii* with *D.natalata* and *D.stellata* seem higher than commonly reported for other Larentiinae genera (Stadie et al. 2014, Wanke et al. 2019, Guerrero et al. 2022). However, sequences deposited in BOLD (Ratnasingham and Hebert 2007) suggest that *Disclisioprocta* harbours several more species than the three recognised until recently (Parsons et al. 1999). Accordingly, it is highly probable that, with an improvement in the knowledge of the taxonomic diversity of *Disclisioprocta*, the genetic distance of *D.edmondsii* to other congenerics would be smaller. Alternatively, further studies could reveal *D.edmondsii* as a member of another, either described or undescribed, lineage of generic level, sister to *Disclisioprocta*. In the meantime, its placement in this genus seems a better solution than its previous adscription to *Xanthorhoe*, in spite the deep genetic distance with congenerics.

Recent molecular phylogenetic analyses clustered *Disclisioprocta* with the Neotropical *Ptychorrhoe* Warren, 1900 in a clade sister to Euphyiini (Brehm et al. 2019, Murillo-Ramos et al. 2019). Unfortunately, the genitalia of *Ptychorrhoe* remain unknown, impeding comparisons with *D.edmondsii*. Further morphological and molecular phylogenetic studies involving members of the Euphyiini + Xanthorhoini complex (Brehm et al. 2019) are encouraged to determine the circumscription of the genera, identifying their synapomorpies and improving the current understanding of the evolution of Larentiinae.

## Supplementary Material

XML Treatment for Disclisioprocta edmondsii
